# The Interface of Nutritional Practices of Selected Basketball Players of Nnamdi Azikiwe University, Awka, On Performance

**DOI:** 10.5539/gjhs.v4n5p192

**Published:** 2012-08-31

**Authors:** Alagbu Chukwubikem Eugene, E. O Agwubuike

**Affiliations:** 1 Department of Human Kinetics & Health Education, Faculty of Education, Nnamdi Azikiwe University, Awka, Nigeria; 2 Department of Health, Environmental & Human Kinetics, Faculty of Education, University of Benin, Benin City, Nigeria

**Keywords:** nutritional practices, athletes, Nnamdi Azikiwe University

## Abstract

The nutritional practices of athletes are critical to sports performance, since good result is the goal or expectations of all sports stake-holders, coaches, sports administrators/managers and spectators alike, therefore the issue of good nutrition regarding these “human machines” (athletes), calls for serious attention. This research, therefore tried to examine the nutritional practices of some selected Basketball players of Nnamdi Azikiwe University (UNIZIK) Awka, in Anambra State of Nigeria. Some 59 male and female Basketball (B/B) players were purposefully selected to participate in the study. A self developed questionnaire (r = 0.71) was administered on them and Weighted Mean Score (WMS). This was in an attempt to ascertain whether the dietary manipulations as practiced by these athletes immediately before competition, affect their performance, in any way. Findings revealed daily inadequate consumption of required proportion of nutrients and very poor timing of meals by the players.

## 1. Introduction

Just before the French Revolution, Lavoisier and the Physicist Laplace carried out experiments in which they placed a guinea-pig in a very small closed chamber, surrounded by ice. They measured the amount of ice that melted over a 10-hour period and at the same time the amount of carbon dioxide given out by the animal. They were able to establish and demonstrate that there was a relationship between the heat produced by the animal and the respiratory exchange. Lavoisier also measured the oxygen consumption of men, and showed that it increased after food and exercise. Hence this study could be said to be apt since the nutritional intake of athletes is said to be critical determinant of the performance and ability to compete both physically and mentally (Economos, Bortz, & Nelson, 2006; Anderson & Young, 2005; Frankl, 2003; Insel & Roth, 2000; Brooks, Fashey, White, & Baldwin, 2000; Burke, 1995; Lewis, 1984). Many individuals who are involved in sports performance now address the dietary requirements of athletes, especially those who are to perform at high metabolic rates for extended periods of training and competition, due to the fact that diet affects metabolism, during rest and physical activity and equally because dietary manipulations has been used to enhance performance in several types of athletes endeavours.

Brooks et al. (2001) stated that no greater mythology exists perhaps in sports than that built around the subject of nutrition. Brooks et al. (2001) stressed that to gain an immediate competitive edge, athletes have engaged in all sorts of odd dietary practices and habits-which if at the end of the day, the athlete becomes successful, he or she continues with the habit or practices, even where the success (unknown to the athlete) may be due to other factors such as genetic endowment, trainings etc.

Again studies have also revealed that the extreme dietary manipulations practiced by some athletes immediately before competitions are more likely to affect performance negatively than positively (Anderson & Young, 2005; Frankl, 2003; Brook et al., 2001; Lewis, 1984). For example, Anderson and Young (2005) warned that eating sugar or honey just before an event, which is commonly practiced by some athletes, does not provide extra energy until 30 minutes after consumption, and that the practice may hinder performance. Anderson and Young (2005) and Frankl, (2003) equally insisted that there is no evidence that megadoses of vitamins or minerals beyond the normal quantity that is obtained by eating mere variety of nutritious foods, improve performance.

Although, less than optimal levels of these micronutrients hinder performance Hawley & Burke (1998). Furthermore Hoffman & Coleman (1991), Hoolkooper (1992), Wardlaw & Insel (1996), all agree that the bottom line of nutritional strategy during competition must include consumption of between 50 – 300gms of carbohydrate (CHO) and a very modestate amount of protein 2 – 6 hrs prior to an endurance event. They therefore listed the food to be avoided during competition session to include:- food known to produce intestinal gas (food rich in fibre and legumes) and hishly seasoned foods. Fox et al. (1979), Girandole et al. (1979) both advocate that athletes who regumarly experience muscle cramp, nausea, diarrhea, indifestion should be placed on moderate liquid pregame meal/food. Increase in caloric intake through a varied/balanced diet ensures sufficient amount of vitamins and minerals for the athlete.

The above cited and other literatures have shown that what really allows an athlete to perform up to his or her potential in training and competition may not be the extreme dietary manipulations practiced by some athletes, but due to sound nutritional practices of what to eat, how much to eat and when to eat.

Anderson & Young (2005), Burke (1995) and Lewis (1984) all agree that no particular food stuff has been identified as possessing the potentialities to make a mediocre/moderately conditioned athlete, to become sports Champion over night. Though it is factual to say that good nutrition plays significant role in an athletes’ performance; the approach as advocated by Brooks et al. (2000), is that adequate diet that is appropriate for the general public (population), is as well appropriate for athletes.

A balanced (or adequate) diet must involve at least three meals per day, in which the daily protein content must be 10-15% of the total energy, carbohydrate content is 55-56% and fat consumption is 30% or less (Fahey et al., 2001; Brooks et al., 2000). The key dietary concerns of every athlete and their handlers must be to meet the athletes increased energy performance requirement and throughout the day to remain fully hydrated.

Adequate nutrition is based on combining specific numbers of servings of foods chosen from each of the four good food groupings in order to receive all the required nutrients. These four food groups according to Howley and Franks (1992) and Getchell, Pippen and Varnes (1987) are:-

- Bread and Cereals group
- Vegetable and Fruit group
- Milk and Milk products group; and
- Meat, Fish, Poultry and Beans group

Apart from good selection and combinations of foods in an athletes diet, proper timing of athletes’ diet before, during and after performance is another nutritional consideration that must be highly valued (Frankl 2003; Fahey et al., 2001).

Presently there are reports that many athletes do not achieve nutritional practices/habits to optimize their sports performance (Economos et al., 2006; Burke, 1995). The factors identified to be responsible for this include poor nutritional knowledge, dietary extremism, poor practical skills in choosing or preparing meals, and reduced access to food due to a busy life style of students of UNIZIK; most of whom combine their studies with search/purchase and preparation of their daily food requirements. Economos et al. (2006) also state that there are limited scientific data about the nutritional practices of athletes, hence this study will in a way shade some light on the nutritional practices of UNIZIK athletes (basketball players). It is not however clear whether athletes follow nutritional recommendations and maintain nutritionally sound diet practices or not. This study was therefore designed, to assess the nutritional habit/practices of selected active Basketball players in UNIZIK Awka.

Answers were sought for the following questions in this research:

1. Do athletes’ meals contain required nutrients?
2. Do athletes regularly observe the minimum three meals per day?
3. Do athletes observe proper timing of their meals?
4. What fluid do the athletes ingest during training/performance?

## 2. Method

The participants were 59 Basketball Players randomly selected from different faculties/departments of UNIZIK Awka, Faculty of Engineering (29); Education (18); Law (4); Social Sciences (4); and Management Sciences (4) using 48 (81.4%) of these athletes who were males, while 11 (18.6%) of them were females. However help were rendered by the research assistants top those that needed assistance/clarifications.

Their mean age was 18.34 ± 5.59 within the range of 18-29 years and their mean years of experience in Basketball was 6.86 ± 7.32 within the range of 1-12 years.

### 2.1 Instrumentation

The main instrument for data collection in this study was a self developed questionnaire. The questionnaire was in three, sections viz (A.B & C) section A dealt with the demographic data of the selected athletes. Section B sought for information on food items consumed and frequency of consumption, while section C focused on meal adequacy, timing and meal during performance, (and source/mode of food preparations).

The instrument was given to three lecturer colleagues for the purpose of validity and their critical contributions and suggestions were considered in the final draft. This instrument was also subjected to test-retest method of reliability test, and its Pearson’s Product Moment Correlation Coefficient (PPMC) result was 0.71.

### 2.2 The Administration of Instrument

The researcher used trained research assistants, who visited these athletes in their training sessions to seek their consents and that of their coach to participate in the research study.

Thereafter copies of the questionnaire were given to them to fill. The research assistants tried to prevent interference among the participating Basketball players as much as possible. However, assistance was rendered by the research assistants to those that needed assistance/clarifications. All the 59 copies of the questionnaire were retrieved immediately after the athletes had completed them.

### 2.3 Data Analysis

The data were coded and analyzed using percentages and WMS because it suits the nature of questionnaire items used in the research work. The component Bars Chart was equally used to give further analyzed description of results of the responses of the respondents.

## 3. Results

The result showed in the table 1 above that the average WMS (3.03) was greater than 2.00 set for high frequency of consumption. It could be seen that food items of bread/cereals group has the highest rating of (3.95) and those of the milk/milk products rated lowest (1.88); which was lower than the criterion value.

**Table 1:** WMS of data of frequency and types of diet consumed by UNIZIK athletes

Food Group	Everyday	1-3 times Per week	Occasionally	Rarely	Not at all	(WMS)
Bread/Cereals	56	03				*3.95
Vegetables/fruits	07	23	24		05	*2.54
Milk/milk products	08	09	12	28	02	1.88
Meat/fish/poultry/beans	47	08	04			*3.73
Average	29.50	10.75	10.00	8.25	0.50	*3.03

**Figure 1:**
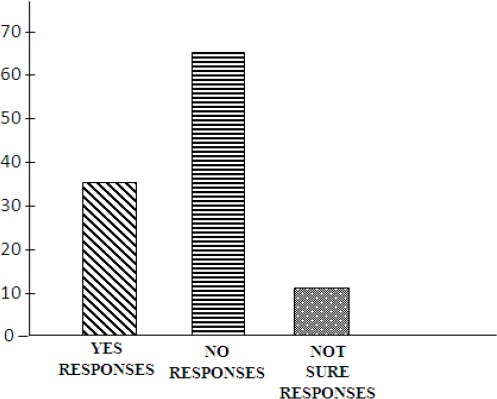
Component bar chart on players’ consumption of three minimum meals per day

From the result presented in table 2 shows that majority of the B/B players (66%) do not take the required minimum of three meals per day. Further more Figure 1 portrays this result vividly.

**Table 2:** Frequency and percentage analysis on minimum meal per day consumed by athletes

Item	Yes (%)	No (%)	Not sure (%)	Total
Takes breakfast, Lunch & Dinner everyday	13(22%)	36(66%)	07(12%)	59 (100%)

**Figure 2:**
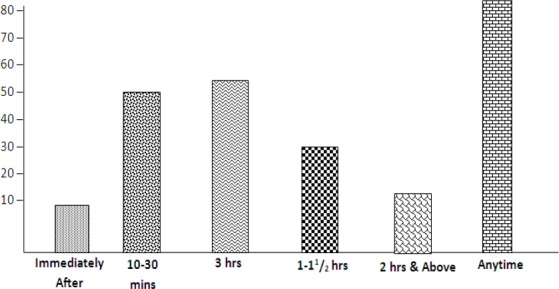


Result in the table 3 above shows that greater percentage of UNIZIK B/B players take their meals anytime before (55.9%) and anytime after (66.1%), trainings and competitions. Only a very minute number of the players (3.4%) observe proper timing of their meals before and after training and competitions. The comparism is depicted in Figure 2 below:

**Table 3:** Frequency and percentage analyses on timing of meals

Timing	Immediately	10-30 Mins	3 hrs %	1-1½ hrs %	≤2hrs %	Anytime %	Total %	Remarks
Meal before performance	01 (17) %	03 (5.1%)	13 (22%)	07 (11.9%)	02 (3.4%)	33 (55.9%)	59 (100%)	
Meal after performance	01 (1.7%)	11 (18.6%)	02 (34%)	05 (8.5%)	01 (1.7%)	39 (66.1%)	59 (100%)	

**Figure 3:**
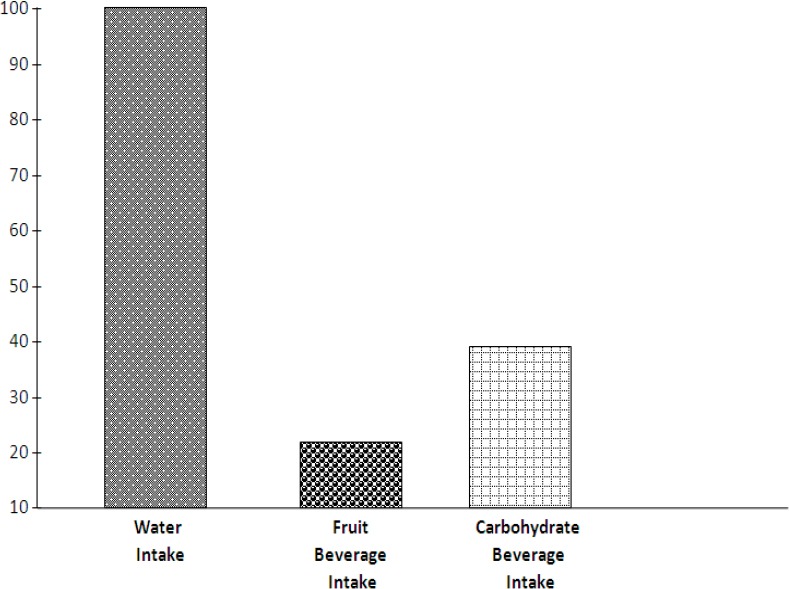
Component bar chart on fluid intake during performance

The result in table 4 above shows that all the players take water during training and competition and just a few among them indicated that in addition to water they also take carbohydrate beverage (13.6%) and fruit Juice (3.4%). The component bar chart also portrays this result more vividly in figure 3.

**Table 4:** Frequency and percentage analysis on fluid ingest during performance

Variables	Water (%)	Fruit Juice %	Carbohydrate Beverages (%)	Remarks
Fluid ingest during training and performance/competition	59(100%)	02 (3.4%)	08 (13.6%)	

## 4. Discussion

The findings of this research revealed that except for foods in the class of Milk and Milk products group, the UNIZIK Basketball players responses to the frequency of their selection of adequate diet is fairly good, (see table 1), however the level of consumption is grossly inadequate as most of these players indicated that they do not take minimum of three meals per day. See also table 2 and figure 1. Equally these practices would definitely have negative effect on the performance level of the players during training and during competitions. Other studies have also shown that athletes must ensure that they have adequate nutrition, which is based on minimum of three meals per day, of four good food groups (Howley & Franks, 1992; Getchell et al., 1987) furthermore the combination of foods should give 55-65% carbohydrate, 10-15% protein, and 30% or less fat (Fahey et al., 2001; Brooks et al., 2000; Lewis, 1984).

Also Fahey et al. (2001) categorically stated that one of the key dietary concerns for athletes is to meet their increased energy requirements, and this can only be ensured through adequate nutrition.

Analysis on proper timing of meal during training and competition by athletes revealed that the greatest percentage of the athletes take their meal anytime before and after competitions (see table 3 and figure 2) nutritionally this is considered as poor practice which affects the performance of athletes negatively. Frankl (2003) opines that food high in complex carbohydrates must be consumed not later than 4 hours before competition since this affects performance in any endurance sports, like Basketball. Frankl (2003) also suggest light carbohydrate-rich meal 15-30 minutes before and heavier meal in about one hour after soccer training sessions or match. However Anderson and Young (2005) suggested 3-4 hours for pre-game meal.

Further findings of this study revealed that what is the most frequently ingested fluid by these B/B players during training and competitions is water, which is in line with what (Frankl, 2003; Fashey et al., 2001; Howley & Franks, 1992; Lewis, 1984) said that there is need for athletes to drink lots of water during performance for adequate hydration. Frank (2003) asserts that a water loss exceeding two percent (2%) of body weight will significantly impair endurance performance. Frankl (2003) furthermore suggests the consumption of carbohydrate beverages by athletes during performance. The assertion was based on reports that such beverages enhance performance better than water especially in endurance activity.

In summary this study revealed poor nutritional practices among UNIZIK Awka basketball players.

The findings of this study therefore agree with previous studies that reported poor nutritional practices among athletes (Economos et al., 2006; Anderson & Young, 2005; Brooks et al., 2002; Burke, 1995). Among the factors that were identified as being responsible for these poor practices were, poor nutritional knowledge, dietary extremism, poor practical skills in choosing or preparing meals, since most of these athletes cook/source for their food by themselves due to poverty, or prevailing circumstances.

## 5. Conclusion

This study may serve as a lead way to finding solutions to the long unanswered questions by many Nigerian sports researchers/stakeholders, as to why the universities in Nigeria, unlike their sister institutions in advanced countries, do not serve as the major source of production of elite athletes for the nation, perhaps this may proffer some answers to questions raised by many, why the Nigerian Universities unlike their counterparts in developed countries fail to produce the required or needed nations elite athletes (Alagbu 2010; Alagbu 2010).

These revealed nutritional problems/poor practices of UNIZIK B/B players may be similar to what other Nigerian university athletes in other areas of sports are also facing, such that if the few recommendations the researcher proffers below are carried out, may change the trend of poor performance of an average Nigerian university athlete.

The rightful expectations of most Nigerians that our universities ought to be or should serve as the factory for the production (manufacturing) of the nations elite athletes who would ably represent the country in world sports competitions and win medals; may never be achieved, unless efforts are made to utilize the results of studies researchers conducted in the areas of sports such as this. The sports achievements attained by the advanced countries could be attributed to their utilization/application of the research findings of their sports scientists and administrators over the years.

## Recommendations

1. Part of the training programme of our university athletes should include education, on proper nutritional practices and their relationship nutrition to sports performance; which must address eating strategies and key food and fluid choices, that will help to achieve goals of sound nutrition in sports
2. The universities being centers of academic excellence should organize seminars, workshops and symposia where experts in exercise physiology, sports medicine and sports nutrition ought to be involved, to educate the university athletes. This should constitute part of the regular trainings of all eligible university athletes on a continuous basis.
3. Regularly university athletes or players should be counseled on their nutritional practices during training and when in camp for competitions. Services of professional experts must be employed for periodical nutritional assessment of university players and athletes.
4. Bursary awards should be re-introduced, specifically for promising/talented university athletes, in addition to free and compulsory hostel accommodation for them, in order to constantly check and review their nutritional habits and practices.
5. Further studies should be encouraged or initiated by other sports scientists on this same topic but focused on other sports areas.
6. The Federal Government of Nigeria should cave out of the present Federal Ministry of Education, a research department, whose duties it would be to collate all the yearly, scientific researches carried out in all the Federal/State universities, and select very good ones for implementation. The nation cannot be longing for sports excellence only on the pages of the national dailies, without digging deep into the store house of numerous researches carried out yearly, begging for utilization/implementation/application.
